# Cleaning ability of two irrigating solutions in primary root canals

**DOI:** 10.6026/973206300222491

**Published:** 2026-04-30

**Authors:** P Nihar, Deepthi M, Mrunal Kalambe, Saloni Jain, Saurabh Prasad, Kavimalar Ravichandran

**Affiliations:** 1 Department of Conservative Dentistry and Endodontics, CKS Theja Dental College, Tirupati, DR. N.T.R University of Health Sciences, Vijayawada, Andhra Pradesh, India; 2 Department of Conservative Dentistry and Endodontics, Dayananda Sagar College of Dental Sciences, SM Hills, KS Layout, Bengaluru, India; 3 Department of Pedodontics and Preventive Dentistry Dr. Rajesh Ramdasji Kambe Dental College, Akola, India; 4 Department of Conservative Dentistry and Endodontics, Bharati Vidyapeeth Deemed University Dental College and hospital, Sangli, India; 5 Department of Conservative Dentistry and Endodontics, Career post graduate Institute Of Dental Sciences, Lucknow; 6 Department of Conservative Dentistry and Endodontics, Sri Ramakrishna Dental College and Hospital, Coimbatore, India

**Keywords:** Primary teeth, pulpectomy, root canal irrigation, sodium hypochlorite, normal saline, debris removal

## Abstract

Effective cleaning of primary root canals remains challenging due to complex anatomy and physiological root resorption, limiting the efficacy of mechanical instrumentation alone. Therefore, it is of interest to compare the cleaning ability of 1% sodium hypochlorite and normal saline as irrigating solutions in primary teeth. Forty extracted primary molars were randomly allocated into two groups (n = 20), instrumented using standardized techniques, and irrigated with either sodium hypochlorite or saline, followed by stereomicroscopic evaluation of residual debris in different canal thirds. The sodium hypochlorite group showed significantly lower debris scores at all levels, particularly in the apical third, compared to the saline group (p < 0.05). Thus, we show that 1% sodium hypochlorite provides superior cleaning efficacy and may be preferred for pulpectomy procedures in primary teeth.

## Background:

Pulpectomy is an essential endodontic procedure performed to preserve primary teeth affected by irreversible pulp pathology until their normal exfoliation. Retention of primary teeth plays a crucial role in maintaining arch length, facilitating mastication and speech and guiding the eruption of permanent successors [1]. The success of pulpectomy depends largely on effective elimination of microorganisms, necrotic tissue remnants and dentinal debris from the root canal system before obturation [2]. Endodontic treatment in primary teeth presents unique challenges when compared to permanent dentition. Primary teeth exhibit complex root canal anatomy characterized by thin dentinal walls, ribbon-shaped canals, lateral and accessory canals and frequent apical ramifications [3, 4]. In addition, physiological root resorption and proximity of the developing permanent tooth germ further complicate canal instrumentation and increase the risk of procedural errors [5]. These anatomical and biological factors limit the effectiveness of mechanical instrumentation alone in achieving thorough canal debridement. Chemical irrigation therefore plays a pivotal role in pediatric endodontics by supplementing mechanical preparation. Irrigating solutions assist in flushing out debris, dissolving organic tissue remnants, lubricating canal walls and reducing microbial load within the root canal system [6].

An ideal irrigant should exhibit strong antimicrobial activity, tissue-dissolving capability, biocompatibility and minimal toxicity to periapical tissues [7]. However, no single irrigant fulfills all these requirements, particularly in the context of primary teeth. Sodium hypochlorite is one of the most widely used irrigating solutions in endodontics due to its potent antimicrobial properties and ability to dissolve organic tissue [8]. Numerous studies have demonstrated its effectiveness against microorganisms commonly isolated from infected root canals [9]. Despite these advantages, its use in primary teeth remains controversial because of its cytotoxic potential, unpleasant taste and the risk of accidental extrusion through resorbing apices, which may cause damage to the surrounding tissues or the underlying permanent tooth germ [10, 11]. Consequently, lower concentrations of sodium hypochlorite are often recommended for pediatric endodontic procedures to balance efficacy and safety [12]. Normal saline is frequently employed as an irrigating solution in primary teeth owing to its isotonic nature, biocompatibility and lack of tissue toxicity [13]. It acts primarily as a flushing agent, facilitating the removal of loose debris from the canal lumen. However, saline does not possess inherent antimicrobial activity or tissue-dissolving properties, which may compromise its effectiveness when used as a sole irrigant in infected root canals [14]. Previous studies evaluating the cleaning efficacy of various irrigating solutions in primary teeth have reported conflicting results [15, 16, 17- 18]. While several authors have demonstrated superior canal cleanliness with sodium hypochlorite, others have emphasized the need for safer irrigants in pediatric patients [19]. Therefore, it is of interest to compare the cleaning efficacy of two commonly used irrigating solutions-1% sodium hypochlorite and normal saline-in primary root canals by assessing residual debris at different canal levels.

## Materials and Methods:

## Study design:

This *in vitro* experimental study was conducted to evaluate and compare the cleaning efficacy of two irrigating solutions in primary root canals under standardized laboratory conditions.

## Sample selection:

A total of 40 freshly extracted human primary molars were selected for the study. Teeth were extracted due to physiological exfoliation or orthodontic reasons and collected after obtaining informed consent. Teeth with at least two-thirds of root length remaining were included.

## Inclusion criteria:

[1] Primary molars with intact roots

[2] Absence of internal or external resorption beyond physiological limits

[3] No previous endodontic treatment

[4] No root fractures or cracks

## Exclusion criteria:

[1] Teeth with excessive pathological resorption

[2] Calcified canals

[3] Grossly damaged or fractured roots

## Sample preparation:

Soft tissue remnants and calculus were removed using an ultrasonic scaler. The teeth were disinfected and stored in normal saline until use. Access cavities were prepared using a sterile round bur under water coolant. Working length was determined by inserting a 10 K-file until visible at the apex and subtracting 1 mm.

## Grouping of samples:

The samples were randomly divided into two groups (n = 20):

[1] Group I: 1% Sodium hypochlorite

[2] Group II: Normal saline

## Root canal instrumentation:

Root canals were instrumented using stainless steel hand K-files up to size #35 employing a standardized step-back technique. During instrumentation, canals were irrigated according to the assigned group protocol.

## Irrigation protocol:

Each canal received a total of 5 ml of irrigating solution, delivered using a side-vented needle placed 1 mm short of the working length. Irrigation was performed after each file change to ensure uniform distribution of the solution.

## Sectioning and evaluation:

After instrumentation and irrigation, the roots were grooved longitudinally using a diamond disc and split into two halves. The halves were examined under a stereomicroscope at 20x magnification.

## Debris scoring:

Residual debris was evaluated at the coronal, middle and apical thirds using a standardized scoring system:

[1] Score 1: Clean canal wall with minimal debris

[2] Score 2: Few debris particles

[3] Score 3: Moderate debris covering canal walls

[4] Score 4: Heavy debris accumulation

## Statistical analysis:

Data were tabulated and statistically analyzed using SPSS software. Intergroup comparison was performed using the Mann-Whitney U test, while intragroup comparison among canal thirds was analyzed using the Kruskal-Wallis test. A p-value < 0.05 was considered statistically significant.

## Results:

All forty primary molar root canals included in the study were evaluated for residual debris following instrumentation and irrigation. Debris scores were assessed at the coronal, middle and apical thirds of the root canal system. The results were statistically analyzed to compare the cleaning efficacy between Group I (1% sodium hypochlorite) and Group II (normal saline). Group I demonstrated consistently lower debris scores compared to Group II across all root canal levels. The difference between the groups was statistically significant, indicating superior cleaning efficacy of 1% sodium hypochlorite. Table 1 (see PDF) shows the comparison of mean debris scores between the two groups at the coronal, middle and apical thirds. Group I exhibited significantly lower debris scores at all three levels when compared to Group II. The difference was most pronounced in the apical third, where saline showed the highest debris accumulation. This indicates that sodium hypochlorite was more effective in cleaning the canal walls, particularly in the apical region. Table 2 (see PDF) presents the intergroup statistical comparison using the Mann-Whitney U test. Statistically significant differences were observed between Group I and Group II at all canal levels. The lowest U value and highest Z score were noted in the apical third, confirming a highly significant difference between the two irrigants, favouring sodium hypochlorite. Table 3 (see PDF) shows the intragroup comparison of debris scores across coronal, middle and apical thirds. Both groups demonstrated statistically significant differences among canal levels. In Group I, debris scores increased from coronal to apical third but remained relatively low. In contrast, Group II exhibited a marked increase in debris accumulation toward the apical third, indicating reduced cleaning efficiency of saline in deeper canal regions. Table 4 (see PDF) illustrates the percentage distribution of debris scores at the apical third. In Group I, the majority of samples showed minimal debris (Scores 1 and 2), while none exhibited heavy debris accumulation. Conversely, Group II demonstrated a high percentage of moderate to heavy debris (Scores 3 and 4), highlighting the inferior cleaning ability of saline, especially in the apical region. 1% sodium hypochlorite demonstrated significantly superior cleaning efficacy compared to normal saline. The apical third showed the greatest difference between the groups. The null hypothesis was statistically challenged by the results and will be formally addressed in the discussion section.

## Discussion:

The present *in vitro* study was conducted to evaluate and compare the cleaning ability of two commonly used irrigating solutions-1% sodium hypochlorite and normal saline-in primary root canals. Effective canal debridement is a critical determinant of pulpectomy success, particularly in primary teeth where complex root canal anatomy and ongoing physiological resorption present additional challenges [2, 3]. The findings of the study demonstrated that 1% sodium hypochlorite exhibited significantly superior cleaning efficacy compared to normal saline at all levels of the root canal system, with the most pronounced difference observed in the apical third. Lower mean debris scores were consistently observed in the sodium hypochlorite group across coronal, middle, and apical thirds. This enhanced cleaning ability can be attributed to the well-established tissue-dissolving and antimicrobial properties of sodium hypochlorite, which facilitate effective removal of organic debris and microbial biofilms [9, 20]. In contrast, normal saline acts merely as an inert flushing agent and lacks both antimicrobial action and tissue dissolution capability, thereby limiting its effectiveness as a sole irrigant [14]. The apical third demonstrated the highest debris accumulation in both groups, although the difference between the two irrigants remained statistically highly significant. This observation is consistent with previous findings indicating that sodium hypochlorite is more effective in removing debris from the apical region of primary teeth compared to inert solutions [15, 16]. The increased debris retention in the apical third may be explained by anatomical complexities such as narrow canals, lateral branches, and apical ramifications, which restrict adequate irrigant penetration and mechanical instrumentation [17, 18- 19]. Intragroup analysis revealed a gradual increase in debris scores from coronal to apical thirds in both groups, further supporting the notion that achieving effective apical cleaning remains a significant challenge in pediatric endodontics. The superior performance of sodium hypochlorite observed in this study aligns with existing evidence emphasizing its broad- spectrum antimicrobial activity and ability to disrupt bacterial biofilms even at lower concentrations [20, 21]. These characteristics make it particularly suitable for use in primary teeth, where safety considerations necessitate the use of diluted irrigants. Despite its effectiveness, concerns have been raised regarding the cytotoxic potential of sodium hypochlorite and the risk of extrusion beyond the apex in primary teeth [11, 12]. However, evidence suggests that the use of low concentrations combined with controlled irrigation techniques-such as side-vented needles and placement short of the working length-can significantly reduce the risk of adverse effects [22, 23]. The standardized irrigation protocol followed in the present study may account for the safe and effective use of sodium hypochlorite. Normal saline, although biocompatible and widely available, demonstrated inferior cleaning efficacy. Its inability to dissolve organic tissue and lack of antimicrobial properties limit its role to that of a supplementary irrigant rather than a primary agent for canal debridement [24]. The findings of the present study therefore support the rejection of the null hypothesis, as a statistically significant difference was observed between the two irrigants at all canal levels, particularly in the apical third [15, 16 and 25]. While the *in vitro* design of the study allowed for standardization of experimental variables, certain limitations must be acknowledged. The absence of dynamic biological factors such as blood flow, tissue fluids, and host immune responses may affect the extrapolation of results to clinical scenarios [26]. Additionally, stereomicroscopic evaluation, although effective for assessing debris, does not provide information regarding bacterial viability. Future studies incorporating microbial analysis and advanced imaging techniques, such as scanning electron microscopy, may provide more comprehensive insights into the effectiveness of irrigating solutions [27, 28, 29-30]. Within these limitations, the present study provides strong evidence supporting the use of low-concentration sodium hypochlorite as an effective irrigating solution for achieving optimal cleaning in primary root canals.

## Conclusion:

1% sodium hypochlorite showed significantly superior cleaning efficacy compared to normal saline in primary root canals. Sodium hypochlorite effectively reduced debris at all canal levels, particularly in the apical third. Normal saline, although biocompatible, was less effective as a sole irrigant. Thus, controlled use of low-concentration sodium hypochlorite during pulpectomy procedures in primary teeth is recommended.
